# Unfavorable impact of decreased muscle quality on the efficacy of immunotherapy for advanced non‐small cell lung cancer

**DOI:** 10.1002/cam4.3631

**Published:** 2020-12-10

**Authors:** Naoya Nishioka, Tateaki Naito, Akifumi Notsu, Keita Mori, Hiroaki Kodama, Eriko Miyawaki, Taichi Miyawaki, Nobuaki Mamesaya, Haruki Kobayashi, Shota Omori, Kazushige Wakuda, Akira Ono, Hirotsugu Kenmotsu, Haruyasu Murakami, Koichi Takayama, Toshiaki Takahashi

**Affiliations:** ^1^ Division of Thoracic Oncology Shizuoka Cancer Center Shizuoka Japan; ^2^ Department of Pulmonary Medicine Graduate School of Medical Science Kyoto Prefectural University of Medicine Kyoto Japan; ^3^ Division of Clinical Research Management Office Shizuoka Cancer Center Shizuoka Japan

**Keywords:** muscle quality, muscle quantity, myosteatosis, non‐small cell lung cancer, PD‐1/PD‐L1 inhibitor

## Abstract

**Background:**

Quantitative skeletal muscle mass loss has the potential to predict the therapeutic effects of immune checkpoint inhibitors. This study aimed to assess the impact of muscular quality on the abovementioned outcomes.

**Methods:**

This study retrospectively reviewed the medical records of patients with advanced non‐small cell lung cancer (NSCLC) who had received PD‐1/PD‐L1 inhibitor monotherapy between March 2016 and February 2018. High muscle quality was stipulated as a skeletal muscle density ≥41 and ≥33 Hounsfield units in patients with a body mass index (BMI) <25 kg/m^2^ and ≥25 kg/m^2^, respectively, as assessed using lumbar computed tomography images. High muscle quantity was stipulated as a lumbar skeletal muscle index ≥41 cm^2^/m^2^ in women, ≥43 cm^2^/m^2^ in men with a BMI <25 kg/m^2^, and ≥53 cm^2^/m^2^ in men with a BMI ≥25 kg/m^2^. We evaluated the associations of these muscular parameters with the overall response rate (ORR), progression‐free survival (PFS), and overall survival (OS).

**Results:**

Out of 156 patients, 80 (51.3%) and 47 (30.1%) showed low muscle quality and quantity, respectively. Patients with high muscle quality showed higher ORR (35.0 vs. 15.8 %, *p*<0.05) and longer PFS durations (median, 4.5 vs. 2.0 months, *p*<0.05) than those with low muscle quality. There were no noted differences in the ORR or PFS between patients with high and those with low muscle quantities. On the contrary, regardless of muscle quality and quantity, there were no differences in OS between patients with high and those with low muscle status.

**Conclusions:**

Lumbar skeletal muscle quality has the potential to predict the therapeutic effect of anti‐programed cell death 1/programed cell death ligand 1 inhibitor monotherapy in patients with advanced NSCLC.

## Graphical abstract

High muscle quality correlates with higher objective response rate and longer progression‐free survival duration in treatment with immune checkpoint inhibitors. On the other hand, there is no association between muscle quantity and the efficacy of immune checkpoint inhibitors.

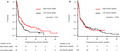

## 1 BACKGROUND

Immune checkpoint inhibitors exert a therapeutic effect under any condition with an appropriate balance between tumor immunogenicity and host immunity in patients with cancer.^1^ Recent reports on the predictors of the therapeutic effects of immune checkpoint inhibitors predominantly focused on tumor antigenicity^2^, ^3^ and the effect of regulatory or suppressor molecules on tumor cells.^4^, ^5^ There are no data on clinically useful predictors for host antitumor immunity.^6^, ^7^ Recently, we proposed a hypothesis regarding the suppressive effects of cancer cachexia on PD‐1/PD‐L1 inhibitors in patients with metastatic non‐small cell lung cancer (NSCLC).^8^ Patients with cachexia exhibited worse overall response rate (ORR) and progression free survival (PFS) values than those without it, even if they were potentially sensitive to anti‐programed cell death 1/programed cell death ligand 1 (PD‐1/PD‐L1) inhibitor and have high PD‐L1 expression in cancer cells. Impaired nutritional status^9^ potentially attenuated the host antitumor immunity in a preclinical study, and several other studies that measured the bodyweight of patients with NSCLC supported our findings.^10^, ^11^

However, results on the predictive impact of quantitative or qualitative skeletal muscle loss, which are hallmarks of cancer cachexia, are inconsistent.^12^ Several articles, including ours,^13^ have reported that decreased muscle quantity, as evaluated by computed tomography (CT), is correlated with poor clinical outcomes in patients who use a PD‐1/PD‐L1 inhibitor.^13^, ^14^, ^15^, ^16^ However, the definitions and measurement methods of muscle depletion varied across studies,^14^, ^15^, ^16^, ^17^ and the association was not always positive.^17^ While some studies reported a negative correlation between muscle quality and the therapeutic effect of PD‐1/PD‐L1 inhibitors,^14^, ^17^ the results were not definitive. Besides, the majority of these studies estimated the impact of muscle quantity or quality without adjusting for previously reported factors, including PD‐L1 expression and pretreatment performance status (PS).

Accordingly, the present study aimed to evaluate whether the quantitative or qualitative loss of lumbar skeletal muscle is predictive of the efficacy of PD‐1/PD‐L1 inhibitors, regardless of confounding factors, in patients with advanced NSCLC.

## 2 METHODS

Consecutive patients with pretreated advanced NSCLC who had undergone PD‐1/PD‐L1 inhibitor monotherapy between March 2016 and February 2018 in Shizuoka Cancer Center were evaluated. The eligibility criteria were as follows: (a) histologically proven stage III or IV NSCLC, including postoperative recurrence, with reference to the seventh or eighth edition of tumor–node–metastasis staging^18^, ^19^; (b) Eastern Cooperative Oncology Group (ECOG) PS0‐2; (c) presence of at least one measurable lesion; (d) prior treatment history with at least one chemotherapy regimen before PD‐1/PD‐L1 inhibitor therapy use; (e) no prior immunotherapy; and (f) availability of evaluable lumbar CT images taken within 1 month before the initiation of PD‐1/PD‐L1 inhibitor therapy. Patients with pretreatment interstitial lung disease were defined as ineligible for this study.

## 3 Data collection

The following data were collected from the medical records of eligible patients: age, sex, PS just before the initiation of PD‐1/PD‐L1 inhibitor therapy, histology, stage, PD‐L1 tumor proportion score (TPS) and driver oncogenes when available, number and types of prior treatments, and the type of PD‐1/PD‐L1 inhibitor received. Pretreatment and previously measured body weight values (measured 6 months before the treatment) were collected, and the body mass index (BMI) and weight changes were calculated. Moreover, neutrophil‐to‐lymphocyte ratios (NLRs), which reflect inflammation, were collected with blood test data just before the initiation of PD‐1/PD‐L1 inhibitor therapy. NLR was calculated as neutrophils divided by lymphocytes, and a high NLR was stipulated as an NLR ≥5.^20^, ^21^ Cancer cachexia was stipulated as an unintentional weight loss >5% during the preceding 6 months or >2% in patients with a BMI <20 kg/m^2^, or the presence of muscle depletion according to the consensus criteria.^22^

### 3.1 Driver oncogenes and PD‐L1 analysis

The status of ALK translocation and EGFR mutation was assessed in all patients with non‐squamous NSCLC, while the BRAF mutation and ROS‐1 translocation status was assessed in some patients, as appropriate. The PD‐L1 TPS values were calculated by trained pathologists using the 22C3 or 28‐8 pharmDx assay (SRL, Inc.a).

### 3.2 Analysis of skeletal muscle

The cross‐sectional area of the lumbar skeletal muscle at the third level of the lumbar vertebra (L3) was analyzed using electronically stored CT scans that were taken before the initial administration of PD‐1/PD‐L1 inhibitors, with or without contrast enhancement within a 5‐mm slice thickness. Lumbar skeletal muscles were identified based on Hounsfield unit (HU) thresholds of −29 to +150.^23^ All analyses were performed using slice‐O‐matic software v5.0 (Tomovision). Muscle quality was calculated as the mean value of the muscular density (HU) in two consecutive images of the cross‐sectional skeletal muscle area at the L3 level.^12^ High muscle quality was stipulated as a skeletal muscle with density ≥41 HU and ≥33 HU in patients with a BMI <25 and ≥25 kg/m^2^, respectively (see Table S1).^12^ Muscle quantity was reported as the lumbar skeletal muscle index (cm^2^/m^2^) and calculated as [cross‐sectional area of skeletal muscle (cm^2^)/height^2^ (m^2^)].^12^ High muscle quantity was defined as a lumbar skeletal muscle index ≥41 cm^2^/m^2^ in women, ≥43 cm^2^/m^2^ in men with a BMI <25 kg/m^2^, and ≥53 cm^2^/m^2^ in men with a BMI ≥25 kg/m^2^.^12^

### 3.3 Statistical methods

The primary outcome was PFS, which was defined as the period between the first day of PD‐1/PD‐L1 inhibitor administration and the day of tumor progression or death, whichever occurred first. The final follow‐up was on July 3, 2019. The secondary outcome was overall survival (OS) and ORR. We defined OS as the period between the first day of PD‐1/PD‐L1 inhibitor administration and the day of death from any cause. Median PFS and OS were assessed by the Kaplan–Meier method. Data for patients without disease progression or who were lost to follow‐up were censored at the time of the last CT scan for PFS assessment. For PFS and period of response, data for patients in whom treatment with new anticancer agents were initiated patients in whom treatment with new anticancer agents were initiated without recognizing tumor progression were censored on the day of the final tumor assessment before treatment with new anticancer agents was initiated. Data for patients who were alive or lost to follow‐up were censored for OS when they were last known to be alive. Log‐rank tests were used to compare the PFS and OS curves. According to the Response Evaluation Criteria in Solid Tumors, ORR was measured as the ratio of the sum of complete response and partial response. Potential predictors were assessed using Cox proportional hazards models for PFS. For the crude hazard ratio, the covariates included PD‐L1 expression (TPS ≥50% vs. <50% or unknown), ECOG‐PS (0–1 vs. 2), muscle quality (high vs. low), muscle quantity (high and low), NLR (≥5 vs. <5), and number of prior treatments (1 vs. ≥2 regimens). The hazard ratio for muscle quality was adjusted for PD‐L1 expression and ECOG‐PS. Fisher’s exact test and the Wilcoxon test were performed to compare various characteristics between the low and high muscle quality groups. All *p*‐values were two‐sided, and a *p*‐value <0.05 was considered statistically significant. All statistical analyses were performed using R.^24^ We analyzed the data using the statistical package R version 3.6.3 (https://cran.r‐project.org/src/base/R‐3/).

## 4 RESULTS

### 4.1 Patient characteristics

**FIGURE 1:**
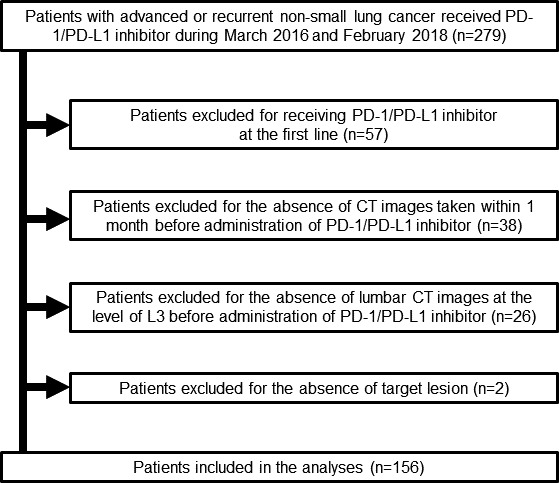
Flow diagram of patient enrollment. Two hundred and seventy‐nine patients were enrolled in this study. However, 123 patients were finally excluded for the following reasons: 2 patients had no measurable lesion, 57 patients received PD‐1/PD‐L1 inhibitors as the first‐line treatment, 26 patients had no CT images evaluable for lumbar skeletal muscle before PD‐1/PD‐L1 inhibitor administration, and 38 patients had no CT within 1 month before PD‐1/PD‐L1 inhibitor administration. PD‐1, programed cell death 1; PD‐L1, programed death‐ligand 1; CT, computed tomography

Out of 279 consecutive patients with advanced NSCLC who initially received PD‐1/PD‐L1 inhibitor therapy between March 2016 to February 2018, 156 were finally enrolled in this study (Figure 1). Reasons for ineligibility included the following: (a) reception of PD‐1/PD‐L1 inhibitors as the first‐line treatment (n = 57), (b) CT was not performed within 1 month before PD‐1/PD‐L1 inhibitor initiation (n = 38), (c) CT did not include the L3 level (n = 26), and (d) patients had no target lesion (n = 2). The patients' median age was 67 (range, 33–85) years (Table 1). The majority of the patients were men, had a PS of 0–1, and had a non‐squamous histology without specific driver oncogenes. Seventy‐four (47.4%) and 82 (52.6%) patients had received PD‐1/PD‐L1 inhibitor therapy as the second‐ and third‐line or greater treatment regimen, respectively. All patients had initiated PD‐1/PD‐L1 inhibitors as monotherapy, including nivolumab in 75 (48.1%) patients, pembrolizumab in 61 (39.1%) patients, and atezolizumab in 20 (12.8%) patients. The pretreatment PD‐L1 TPS was measured in the tissue samples of 109 (69.9%) patients and classified into the following three groups: (a) TPS of ≥50% in 36 (23.1%) patients, (b) TPS between 1% and 49% in 36 (23.1%) patients, and (c) TPS of 0% in 37 (23.7%) patients. Either the PD‐L1 level was not measured or the tumor samples could not be used in evaluating the PD‐L1 TPS in 47 (30.1%) patients who were categorized as having an unknown PD‐L1 status. Sixty‐seven (43.0%) patients had a diagnosis of cancer cachexia at baseline. Eighty (51.3%) and 48 (30.8%) patients had a high muscle quality and quantity at the baseline, respectively. No significant differences were found regarding characteristics between patients with high and those with low muscle quality values, except for median age (65.5 vs. 69 years, *p *< 0.05) and mean attenuation (45.0 ± 4.74 vs. 32.8 ± 5.13 HU, *p *< 0.05). Out of patients with a high muscle quality, 30 (39.5%) and 58 (72.5%) patients had cancer cachexia and a low muscle quantity, respectively.

**TABLE 1:** Patients characteristics

Characteristics		Total (n = 156)	High muscle quality^e^ group (n = 80)	Low muscle quality group (n = 76)	*p*‐value
Age median (range)		67 (33–85)	65.5 (33–81)	69 (44–85)	*p* < 0.05
Gender (%)	Men	101 (64.7)	55 (68.8)	46 (60.5)	NS
	Women	55 (35.3)	25 (31.2)	30 (39.5)	
ECOG‐PS (%)	0	24 (15.4)	12 (15.0)	12 (15.8)	NS
	1	115 (73.7)	63 (78.8)	52 (68.4)	
	2	17 (10.9)	5 (6.2)	12 (15.8)	
Histology (%)	Squamous	22 (14.1)	10 (12.5)	12 (15.8)	NS
	Non‐squamous	134 (85.9)	70 (87.5)	64 (84.2)	
Stage (%)	III	33 (21.2)	17 (21.2)	16 (21.1)	NS
	IV	123 (78.8)	63 (78.8)	60 (78.9)	
Driver oncogene (%)	EGFR mutation	27 (17.3)	12 (15.0)	15 (19.7)	NS
	ALK rearrangement	1 (0.6)	0 (0.0)	1 (1.2)	
	Others^a^	128 (82.1)	68 (85.0)	60 (78.9)	
PD‐L1 Tumor proportion score (%)	≥50%	36 (23.1)	21 (26.2)	15 (19.7)	NS
	1~49%	36 (23.1)	19 (23.8)	17 (22.4)	
	0%	37 (23.7)	16 (20.0)	21 (27.6)	
	Unknown	47 (30.1)	24 (30.0)	23 (30.3)	
Number of prior treatment (%)	1	74 (47.4)	44 (55.0)	30 (39.5)	NS
	≥2	82 (52.6)	36 (45.0)	46 (60.5)	
Initial PD‐1/PD‐L1 inhibitor (%)	Nivolumab	75 (48.1)	37 (46.2)	38 (50.0)	NS
	Pembrolizumab	61 (39.1)	34 (42.6)	27 (35.5)	
	Atezolizumab	20 (12.8)	9 (11.2)	11 (14.5)	
BMI (kg/m^2^, mean ± SD)		21.9±3.79	21.3±4.32	22.5±3.07	NS
Neutrophil‐to‐lymphocyte ratios^b^	High (≥5)	35 (22.4)	18 (22.5)	17 (22.4)	NS
	Low (<5)	121 (77.6)	62 (77.5)	59 (77.6)	
LSMI (cm^2^/m^2^, mean ± SD)		41.0±7.80	41.6±8.44	40.3±7.07	NS
Mean attenuation (HU, mean ± SD)		39.0±7.85	45.0±4.74	32.8±5.13	*p* < 0.05
Pretreatment cachexia^c^ (%)		67 (43.0)	30 (39.5)	37 (46.2)	NS
Pretreatment muscle quantity^d^ (%)	High	47 (69.9)	22 (27.5)	25 (32.9)	NS
	Low	109 (69.9)	58 (72.5)	51 (67.1)	NS

Abbreviation; ECOG‐PS, Eastern Cooperative Oncology Group performance status; PD‐L1, programed cell death protein ligand 1; PD‐1, programed death‐1; BMI, body mass index; ns, not significant; EGFR, Epidermal Growth Factor Receptor; ALK, Anaplastic Lymphoma Kinase; HU, Hounsfield Unit; SD, Standard deviation.

^a^ Others include all patients with squamous cell carcinoma and patients with non‐squamous carcinoma without specific driver oncogenes (EGFR mutation or ALK rearrangement).

^b^ Neutrophil‐to‐lymphocyte ratios were collected with blood test data just before the initiation of PD‐1/PD‐L1 inhibitor therapy. Neutrophil‐to‐lymphocyte ratios were calculated as neutrophils divided by lymphocytes and high NLR was stipulated as ≥5.

^c^ Cancer cachexia was defined as an unintentional weight loss >5% during the preceding 6 months or >2% in patients with a BMI <20 kg/m^2^, or the presence of muscle depletion according to consensus criteria.

^d^ High muscle quantity was defined based on lumbar skeletal muscle index cutoffs ≥43.0 cm^2^/m^2^ for men with a BMI <25.0 kg/m^2^, ≥53.0 cm^2^/m^2^ for men with a BMI ≥25.0 kg/m^2^, and ≥41.0 cm^2^/m^2^ for women.

^e^ High muscle quality was defined based on lumbar skeletal muscle index cutoffs of ≥41.0 HU in those with a BMI <25.0 kg/m^2^, and ≥33 HU in those with a BMI ≥25.0 kg/m^2^.

### 4.2 Impact of muscle quality and quantity on tumor response

The ORR was 25.6% in all patients (95% confidence interval [CI], 1.26–6.80). Patients with high tumor PD‐L1 expression (TPS ≥50%) had a higher ORR than those with low or unknown PD‐L1 expression (50.0% vs. 18.3%, *p *< 0.05). Patients with a high muscle quality had a higher ORR than those with a low muscle quality (35.0 % vs. 15.8%, *p *< 0.05). Conversely, no difference in the ORR was noted between patients with high and those with low muscle quantity values (24.8% vs. 27.7%, *p *= 0.70). We further assessed the response rate for each muscle quality group. There was no difference in the ORR in the high muscle quality group between patients with high and those with low muscle quantity values (40.9% vs. 32.8%, *p *= 0.60). Similarly, in the low muscle quality group, no difference in the ORR was noted between patients with high and those with low muscle quantity values (16.0% vs. 15.7%, *p *= 1.00).

### 4.3 Impact of muscle quality and quantity on progression‐free survival

**FIGURE 2:**
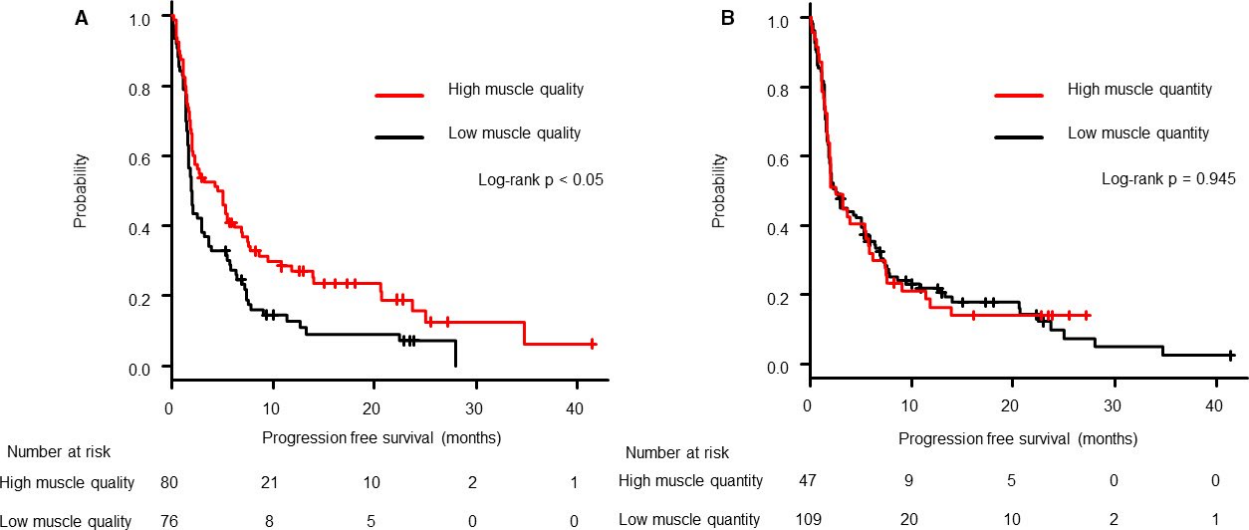
Kaplan–Meier estimates of progression‐free survival in patients classified according to high or low muscle quality (A) and by high and low muscle quantity (B). A greater benefit was observed in patients with high muscle quality showing significantly prolonged progression‐free survival durations. No noticeable difference was observed between the high and low muscle quantity groups

Out of the 156 patients, 133 (85.3%) showed tumor progression up to the cutoff date (July 3, 2019). After a median follow‐up of 2.5 months (95% CI, 2.0–4.2 months), patients with high tumor PD‐L1 expression (TPS ≥50%) had a longer PFS duration than those with low or unknown PD‐L1 expression (6.3 vs. 2.1 months in median, *p *< 0.05). Patients with high muscle quality had a longer PFS than those with low muscle quality (4.5 vs. 2.0 months, *p *< 0.05, Figure 2A). No significant difference in the PFS was noted between patients with high vs. low muscle quantity values (2.5 vs. 2.6 months, *p *= 0.95, Figure 2B). In the multivariate analyses, high muscle quality was significantly associated with superior PFS values, and the adjusted hazard ratio for high muscle quality was 0.67 (95% CI, 0.42–0.89; *p *< 0.05), after adjusting for PD‐L1 expression, pretreatment PS, and number of prior treatments (Table 2).

**TABLE 2:** Predictor for efficacy in PD‐1 /PD‐L1 inhibitors

Predictors for PFS	Crude HR	95% CI	*p*‐value	Adjusted HR ^d^	95%CI	*p*‐value
PD‐L1 expressions (TPS≥50% vs. <50% or unknown)	0.51	0.90–1.79	*p* < 0.05	0.55	0.34–0.89	*p* < 0.05
ECOG‐PS (0–1 vs. 2)	0.57	0.38–0.86	*p* < 0.05	0.65	0.44–0.99	*p* < 0.05
Muscle quality^a^ (High vs. Low)	0.63	0.44–0.89	*p* < 0.05	0.64	0.45–0.92	*p* < 0.05
Muscle quantity^b^ (High vs. Low)	0.98	0.68–1.42	0.94	NI	NI	NI
Neutrophil‐to‐lymphocyte ratio^c^ (NLR≥5 vs. NLR<5)	1.97	1.32–2.94	*p* < 0.05	2.28	1.46–3.56	*p* < 0.05
Number of priory treatment (one vs. ≥2 regimens)	1.27	0.90–1.79	0.18	NI	NI	NI

Abbreviations: CI, Confidence interval; ECOG‐PS, Eastern Cooperative Oncology Group performance status; HR, hazard ratio; NI, not include in the multivariate model; NLR, Neutrophil‐to‐lymphocyte ratio; PD‐L1, programed cell death ligand 1; PFS, progression free survival.

^a^ High and low muscle quality was defined according to muscle attenuation (HU) and BMI. Low muscle quality was defined based on mean attenuation cutoffs <41.0 HU in those with a BMI <25.0 kg/m^2^, and <33.0 HU in those with a BMI ≥25.0 kg/m^2^. There was no difference in the cutoffs between the men and women.

^b^ High and low muscle quantity was defined according to lumbar skeletal muscle index and BMI. Low muscle quantity was defined based on lumbar skeletal muscle index cutoffs <43.0 cm^2^/m^2^ for men with a BMI <25.0 kg/m^2^, <53.0 cm^2^/m^2^ for men with a BMI ≥25.0 kg/m^2^, and <41.0 cm^2^/m^2^ for women.

^c^ Neutrophil‐to‐lymphocyte ratio was defined as neutrophils divided by lymphocytes, and both neutrophil and lymphocyte counts were based on the results of blood collection immediately before administration of PD‐1/PD‐L1 inhibitors.

^d^ HR for PD‐L1 expression was adjusted for muscle quality, ECOG‐PS, and number of prior treatments; HRs for muscle quality and quantity were adjusted for PD‐L1 expression, Neutrophil‐to‐lymphocyte ratio, ECOG‐PS, and number of prior treatments.

### 4.4 Impact of muscle quality and quantity on OS

**FIGURE 3:**
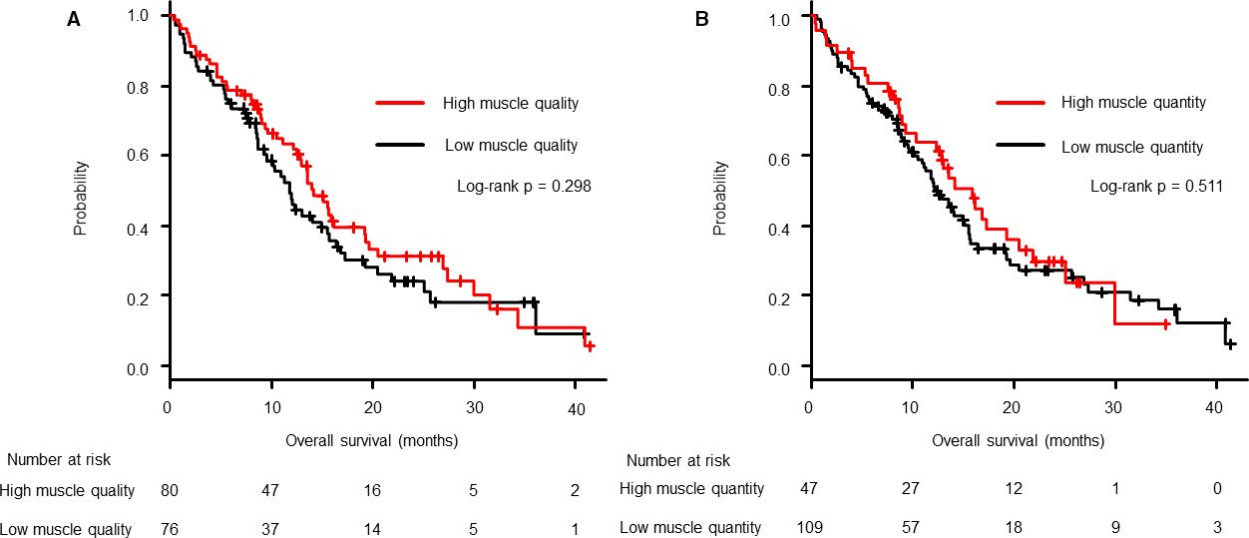
Kaplan–Meier estimates of overall survival in patients classified according to high or low muscle quality (A), and high or low muscle quantity (B). No noticeable difference was observed in the overall survival between the high and low muscle quality groups as well as between the high and low quantity groups

Of the 156 patients, 105 (67.3%) had died before the cutoff date (July 3, 2019). After a median follow‐up of 11.2 (range, 0.4–42) months, no significant difference in OS was noted between patients with high and those with low muscle quality values (14.1 vs. 11.9 months, *p *= 0.30, Figure 3A). Similarly, there was no significant difference in OS between patients with high and those with low muscle quantity values (15.9 vs. 12.2 months, *p *= 0.51, Figure 3B).

## 5 DISCUSSION

This study suggests that lumbar muscle quality can serve as a sensitive predictor of the therapeutic effects of PD‐1/PD‐L1 inhibitor monotherapy in patients with NSCLC. We found that high muscle density, as determined by lumbar CT images taken just before the initiation of PD‐1/PD‐L1 inhibitor therapy, was significantly associated with a high ORR and long PFS duration, irrespective of the degree of PD‐L1 expression in the tumor and PS, both of which are established predictors of efficacy in these treatments. On the contrary, with regard to OS, there was no significant association between the therapeutic effects of PD‐1/PD‐L1 inhibitor and muscle quality or quantity.

Several previous cross‐sectional studies have researched the role of muscle quantity in the prediction of PFS or OS with regard to PD‐1/PD‐L1 inhibitor use in patients with advanced NSCLC. However, the results were inconsistent, with one study reporting the lack of association^17^ and three other studies demonstrating positive associations with PFS or OS (Table 3).^14^, ^15^, ^16^ All of the studies had a retrospective design and included small numbers of patients (23–100 patients). Assessment tools included the cross‐sectional area of the psoas or lumbar muscles divided by body height squared. However, three of these studies did not assess the effects of PD‐L1 expressions on outcomes.^14^, ^15^, ^16^ Only one study adjusted for the predictive impact of muscle quantity by PD‐L1 expression and reported the absence of an association with PFS,^17^ which is consistent with our study, although the assessment tool used for muscle quantity was different. According to the reports mentioned above and the results of our study, the association between muscle quantity and the therapeutic effects of PD‐1/PD‐L1 inhibitor therapy remains controversial and needs further evaluation.

**TABLE 3:** Summary of literatures about the relationship between skeletal muscle quantity or quality and the efficacy of PD‐1/PD‐L1 inhibitors

Publication (year)	Number of patients	Cancer type	Skeletal muscle quantity		Skeletal muscle quality		Adjustment by PD‐L1
			Assessment tool	Association with PFS	Assessment tool	Association with PFS	
Cortellini et al. (2019)	23	NSCLC	LSMI	Yes	NA	NA	No
Shiroyma et al. (2019)	42	NSCLC	PMI	Yes	NA	NA	No
Minami et.al. (2020)	74	NSCLC	PMI	No	IMAC in multifidus muscle	No	Yes
Cortellini et al. (2020)	100	NSCLC and others	LSMI	Yes	Mean density	No	No
Our study	156	NSCLC	LSMI	No	Mean density	Yes	Yes

Previous reports that examined the association between muscle assessment and the efficacy of PD‐1/PD‐L1 inhibitor therapy.

Abbreviation: PD‐L1, Programed death‐ligand 1; NA, Not assessed; NSCLC, Non‐Small Cell Lung Cancer; CT, Computed Tomography; ORR, Overall Response Rate; PFS, Progression Free Survival; OS, Overall Survival; IMAC, intramuscular adipose tissue content; PMI, psoas muscle index; LSMI, lumber skeletal muscle index.

Likewise, while the role of low muscle quality in immunotherapy is currently a hot research topic, no conclusions have been drawn to date. While a previous study stated that decreased lumbar muscle density is predictive of inferior PFS in patients with metastatic melanoma treated with ipilimumab,^25^ several other studies conducted in patients with metastatic NSCLC treated with PD‐1/PD‐L1 inhibitors were skeptical about its predictive value.^10^, ^17^ Cortellini et al. retrospectively reviewed the impact of muscle quality on PFS in association with PD‐1/PD‐L1 inhibitor use in a mixed cancer population comprising 46 patients with advanced NSCLC and 54 patients with other advanced cancers including renal cell carcinoma and melanoma.^14^ They measured lumbar skeletal muscle density as an indicator of muscle quality and showed no association between muscle quality and PFS or ORR. Minami et al. retrospectively reviewed 74 patients with pretreated and advanced NSCLC who had received PD‐1/PD‐L1 inhibitor therapy.^17^ They used the intramuscular adipose tissue content in the multifidus muscle as an indicator of muscle quality and reported the absence of impact on PFS. Unlike the two studies mentioned above, our study found a positive association between muscle quality and PFS and ORR patients with PD‐1/PD‐L1 inhibitor use. The inconsistencies in the results may be attributed to the methodologies used for muscular quality assessment across studies and the heterogeneity of each study population with regard to multi‐factors that were possibly associated with cancer cachexia, including levels of inflammation, complications, or age distribution. Additionally, all previous studies including ours have been categorized as retrospective studies and only included a small number of patients in various treatment lines with a different PS value or driver oncogene status. Thus, we believe that adjustments for established confounding factors, including cancer type,^26^, ^27^ PS,^28^ and PD‐L1 expression^29^ are essential for estimating the impact of muscular quality on PFS. Our study focused on pretreated patients with advanced NSCLC (n = 156) who were already shown to be refractory to standard chemotherapy or targeted treatment and were naïve to immunotherapy. The predictive impact of muscular quality was tested in the univariate and multivariate analyses, after adjusting for PS and PD‐L1 expression. Thus, we propose that muscular quality may be a potential predictor of the therapeutic effects of PD‐1/PD‐L1 inhibitor therapy; our findings require further validation in future prospective studies.

Decreased muscle quality or myosteatosis is usually assessed by CT density and reflects lipid infiltration in degenerative muscle tissue.^30^ Recently, myosteatosis has been recognized as a strong prognostic factor, independently of muscle quantity, in patients with cancer,^12^ and has been shown to be more sensitive to changes in muscle function than muscle quantity in patients with rheumatoid arthritis^30^ or community‐dwelling elderly populations.^31^, ^32^ Therefore, it is possible that our analysis simply reflected the prognostic aspect of muscle quality, and not a predictive aspect. However, we showed significantly higher ORR in the high muscle quality group, and this may support the hypothesis that muscle quality is not only a prognostic factor, but also a predictive factor in immunotherapy. Furthermore, high muscle quality and quantity did not prolong OS in this study, and this result further supports our hypothesis that muscle quality may not be a prognostic factor but a predictive factor.

Although it is unknown why immunotherapy is less efficacious in patients with myosteatosis, several studies have suggested a possible association between myosteatosis, inflammation, and the efficacy of immunotherapy. The presence of myosteatosis has been reportedly associated with elevated inflammatory marker values, including those of tumor necrosis factor (TNF)‐α, interleukin (IL)‐6, the NLR, and C‐reactive protein in humans.^33^, ^34^, ^35^ IL‐6 increases glucocorticoid levels in the serum, which may impair the infiltration and proliferation of CD8+ T cells within the tumor and weaken the therapeutic effects of PD‐1/PD‐L1 inhibitors.^9^, ^36^ TNF‐α not only compromised CD8+ tumor‐infiltrating lymphocytes, but also reduced the degree of PD‐L1 expression on tumor cells in a murine model of melanoma.^37^ The negative effects of IL‐6 on outcomes in cases with PD‐1/PD‐L1 inhibitor use has been shown in a clinical study of patients with melanoma.^38^ Besides, persistent systemic inflammation induces regulatory T cells and myeloid‐derived suppressor cells in tumors, which in turn suppress the levels of antitumor immunocytes, including NK cells, CD8 + T cells, dendritic cells, and B cells, and this eventually promoted tumor progression in experimental models.^39^, ^40^ Furthermore, tumors escape from immune surveillance through the induction of immune checkpoint molecules such as PD‐L1 in tumors and CTLA‐4 receptors in T cells under the influence of chronic inflammation.^41^, ^42^ Therefore, we hypothesize that decreased muscle quality reflects an increased degree of tumor‐associated inflammation, which enhances the resistance against antitumor immunity and results in poor outcomes in PD‐1/PD‐L1‐targeted treatments.

There are some limitations in this study. First, our analysis was limited by its retrospective nature, for which we were unable to exclude biases associated with unknown factors. Second, the small sample size comprises patients from a single institution, limiting our ability to generalize our results to other populations. Third, we did not measure the level of tumor antigenicity by the tumor mutation burden or microsatellite instability because they are not routinely measured in Japanese clinical practice in advanced NSCLC settings. Fourth, there was a possibility that we could not accurately assess the impact of muscle quality on the OS of patients because it did not directly influence the therapeutic effects of PD‐1/PD‐L1 inhibitors in patients with various statuses with regard to PS, treatment line, driver oncogene, and presence of cancer cachexia. Finally, this study did not include a control cohort that did not receive immunotherapy, and therefore, it was difficult to make accurate assessments or conclusions regarding predictive roles. A further prospective study is needed to eliminate these biases and patient heterogeneity for the confirmation of our results.

## 6 CONCLUSIONS

High lumbar skeletal muscle quality may potentially be used to predict the therapeutic effects of PD‐1/PD‐L1 inhibitors, independent of PS and PD‐L1 expression, in patients with advanced NSCLC. However, further studies are needed to validate our results.

## CONFLICT OF INTERESTS

T.N. reported grants from Ono Pharmaceutical Co., Ltd., Pfizer US, Inc., and Mochida Pharmaceutical Co., Ltd. that are outside of the submitted work. H.K. reported grants from Eli Lilly K.K and Taiho Pharmaceutical that are outside the submitted work. S.O. reported grants from Chugai Pharmaceutical Co., Ltd., Ono Pharmaceutical, AstraZeneca K.K., Boehringer Ingelheim, Taiho Pharmaceutical, and MSD that are outside the submitted work. K.W. reported grants from Chugai Pharmaceutical Co., Ltd., Taiho Pharmaceutical, Boehringer Ingelheim, Eli Lilly K.K., Ono Pharmaceutical, and MSD that are outside the submitted work. A.O. reported grants from Taiho Pharmaceutical, Ono Pharmaceutical, Chugai Pharmaceutical Co., Ltd., and Novartis Pharma K.K. that are outside the submitted work. H.K. reported grants from Ono Pharmaceutical Co, Ltd., Kyowa Hakko Kirin Co., Ltd., Bristol‐Myers Squibb, MSD, Eli Lilly K.K, Novartis Pharma K.K., Daiichi‐Sankyo Co., Ltd., and Pfizer K.K., and grants and personal fees from AstraZeneca K.K., Chugai Pharmaceutical Co, Ltd., and Boehringer Ingelheim that are outside the submitted work. H.M. reported grants and personal fees from AstraZeneca, Chugai Pharmaceutical Co., Ltd., Taiho Pharmaceutical, and Eli Lilly Japan., and grants from Abbvie, Daiichi Sankyo, and IQvia, and personal fees from Ono Pharmaceutical, Bristol‐Myers Squibb Japan, and MSD that are outside the submitted work. K.T. reported Lecture fee from AstraZeneca, MSD, Chugai‐Roche Co., Ono Pharmaceutical Co., Eli Lilly, Boehringer‐Ingelheim Co., and grants from Chugai‐Roche Co., Ono Pharmaceutical Co. that are outside the submitted work. T.T. reported grants and personal fees from AstraZeneca KK, Pfizer Japan, Inc., Eli Lilly Japan K.K., Chugai Pharmaceutical Co., Ltd., Ono Pharmaceutical Co., Ltd., MSD K.K., and Boehringer Ingelheim Japan, Inc., and personal fees from Roche Diagnostics K.K. that are outside the submitted work. The other authors have no conflict of interest to declare.

## AUTHORS' CONTRIBUTIONS

Naoya Nishioka and Tateaki Naito Conceptualized the research, designed the study, interpreted the results and wrote the manuscript. Akifumi Notsu and Keita Mori oversaw the analyses as biostatisticians. Hiroaki Kodama, Eriko Miyawaki, Taichi Miyawaki, Nobuaki Mamesaya, Haruki Kobayashi, Shota Omori, Kazushige Wakuda, Akira Ono, Hirotsugu Kenmotsu, and Haruyasu Murakami: Collected data. Koichi Takayama and Toshiaki Takahashi: Revised the final version of the manuscript.

## ETHICS APPROVAL AND CONSENT TO PARTICIPATE

This retrospective cohort study was approved by the institutional review board of Shizuoka Cancer Center, Japan (J2019‐196), and performed under the ethical standards laid down in the 1964 Declaration of Helsinki and its later amendments. The need for informed consent was waived owing to the retrospective nature of the study.

## Supporting information

Table S1

## Data Availability

The data sets generated during and/or analyzed during the current study are available from the corresponding author on reasonable request.
