# Supplementary material for: Unfavorable impact of decreased muscle quality on the efficacy of immunotherapy for advanced non‐small cell lung cancer
Source: Cancer Med. 2020 Dec 10;10(1):247–56. doi: 10.1002/cam4.3631 (PMC7826480; doi:10.1002/cam4.3631)
Supplement: Supplementary file 1 — Table S1 [file CAM4-10-247-s001.docx]

Supplementary Table 1

|  | Sex | Body index mass (kg/m^2^) | |
| --- | --- | --- | --- |
|  |  | <25 | ≥25 |
| LSMI (cm^2^/m^2^) | Male | <43 | <53 |
|  | Female | <41 | <41 |
| Muscle density  (HU) | Male | <41 | <33 |
|  | Female |  |  |

Abbreviation; LSMI, Lumber skeletal muscle index; HU, Hounsfield unit
